# Bacterial and abiogenic carbonates formed in caves–no vital effect on clumped isotope compositions

**DOI:** 10.1371/journal.pone.0245621

**Published:** 2021-01-25

**Authors:** Attila Demény, László Rinyu, Péter Németh, György Czuppon, Nóra Enyedi, Judit Makk, Szabolcs Leél-Őssy, Dóra Kesjár, Ivett Kovács

**Affiliations:** 1 Institute for Geological and Geochemical Research, Research Centre for Astronomy and Earth Sciences, Budapest, Hungary; 2 Isotope Climatology and Environmental Research Centre (ICER), Institute for Nuclear Research, Debrecen, Hungary; 3 Institute of Materials and Environmental Chemistry, Research Centre for Natural Sciences, Budapest, Hungary; 4 Department of Earth and Environmental Sciences, University of Pannonia, Veszprém, Hungary; 5 Department of Microbiology, Eötvös Loránd University, Budapest, Hungary; 6 Department of Physical and Applied Geology, Eötvös Loránd University, Budapest, Hungary; University of Florida, UNITED STATES

## Abstract

Speleothems (dominated by cave-hosted carbonate deposits) are valuable archives of paleoclimate conditions. As such, they are potential targets of clumped isotope analyses that may yield quantified data about past temperature variations. Clumped isotope analyses of stalagmites, however, seldom provide useful temperature values due to various isotope fractionation processes. This study focuses on the determination of the microbially induced vital effect, i.e., the isotope fractionation processes related to bacterial carbonate production. A cave site with biologically mediated amorphous calcium carbonate precitation was selected as a natural laboratory. Calcite deposits were farmed under a UV lamp to prevent bacterial activity, as well as under control conditions. Microbiological analyses and morphological investigations using scanning electron microscopy showed that the UV lamp treatment effectively reduced the number of bacterial cells, and that bacterial carbonate production strongly influenced the carbonate’s morphology. Stable oxygen isotope analyses of calcite and drip waters, as well as clumped isotope measurements revealed that, although most of the studied carbonates formed close to oxygen isotope equilibrium, clumped isotope Δ_47_ values varied widely from equilibrium to strongly fractionated data. Site-specific kinetic fractionations played a dominant role in the distribution of Δ_47_ values, whereas bacterial carbonate production did not result in a detectable clumped isotope effect.

## Introduction

Speleothems are important subjects of paleoclimate research because of the possibility of precise absolute age determination [1] and the wealth of environmental proxies that can be gathered from their analyses at relatively high, sometimes even at sub-annual temporal resolution [2]. Because many of the speleothems are almost pure calcium carbonates, they can be excellent targets for clumped isotope analysis, a technique that is based on preferential bonding of heavy carbon and oxygen isotopes in carbonate, relative to the stochastic distribution of light and heavy isotopes. Clumped isotope analysis can help to determine carbonate formation temperature [3,4]. Isotope clumping is expressed by the Δ_47_ value, which is the relative deviation of the abundance of the mass 47 CO_2_ (largely ^13^C^18^O^16^O) molecule from the stochastic distribution.

Several empirical and experimental equations were published in the last decade, providing slightly different Δ_47_-temperature relationships for biogenic carbonates [5], travertines [6], and experimental carbonates (e.g., [7–9]). Following the approach by Coplen [10], who suggested that the extremely slowly depositing calcite of the Devils Hole (Nevada, USA) may represent true calcite-water equilibrium in a natural environment, a recent study by Daëron et al. [11] offered equations for “equilibrium” temperature dependence of clumped isotope composition and calcite-water oxygen isotope fractionation, based on data gathered for the Devils Hole calcite and a similarly slowly precipitating carbonate deposit formed in the Corchia Cave (Italy). “Equilibrium” is denoted in quotation marks, as true thermodynamic equilibrium in natural carbonate formations becomes ambiguous if empirical observations and theoretical calculations are evaluated together (see theoretical calculations [12] and results for expected natural equilibrium conditions for extremely slowly depositing calcites [11]). In this paper, the term “equilibrium” will be used for the carbonates that yield carbonate-water oxygen isotope fractionation values and Δ_47_ data approaching the isotope-temperature relationships defined by the data of extremely slowly precipitating calcites [11]. In contrast to the Devils Hole and Corchia Cave deposits [11], stalagmites, the most frequently studied speleothems, generally yield Δ_47_ data that significantly deviate from the “equilibrium” values, due to kinetic processes [13,14]. Flowstones, however, are similar to the travertine formations that yield close-to-“equilibrium” Δ_47_ data [6,9]. As a result, a pilot study was begun on speleothems in the Baradla Cave (NE Hungary), where stalagmites and flowstones have been researched [15–17]. At a preliminary stage of this pilot study, the flowstones yielded close-to-“equilibrium” clumped isotope compositions, while the stalagmites’ Δ_47_ data were strongly shifted to lower values. Additionally, it was discovered that bacterial carbonate precipitation produced amorphous calcium carbonate in the Baradla Cave [15,18], which was preserved by a covering of an extracellular polymeric substance (lipids, proteins, carbohydrates, and nucleic acids, [18]). This observation raised the question of whether microbial carbonate production exerted a vital effect on clumped isotope compositions, or whether the low stalagmite Δ_47_ values were attributed solely to abiogenic isotope fractionations. The possibility of a vital effect was further supported by Thaler et al. [19], who detected low Δ_47_ values in microbial carbonates. They attributed the shift in Δ_47_ values (up to –0.27‰) to ureolytic DIC (dissolved inorganic carbon) production in the bacterial cells. This mechanism can be expected in the bacteria thriving in the Baradla Cave, where heterotrophic bacterial strains that precipitate carbonate through ureolysis were identified [18]. The amount of microbial carbonate is estimated to be 10–50% from textural observations and Fourier Transform Infrared Spectroscopy (FTIR) [15,19]. Combining the amount of microbial carbonate and the potential isotope fractionation would suggest an expected Δ_47_ shift of about 0.03–0.1‰, which is above the current analytical precision. To test whether bacterial carbonate production affects the clumped isotope composition of the precipitating carbonate, the Baradla Cave was used as a natural laboratory, where carbonate samples were deposited and collected at one site illuminated by a UV lamp and at a control site that was not treated. The research sites were selected based on previous monitoring activity [15,20]. The carbonate was deposited on glass plates and underwent microbiological analysis, scanning electron microscopy (SEM), Fourier Transform Infrared Spectroscopy (FTIR), micro-X-Ray diffraction (micro-XRD), laser spectroscopy for stable oxygen isotope compositions of drip waters, and stable isotope mass spectrometry for stable carbon, oxygen, and clumped isotope compositions of carbonates.

## Samples and analytical methods

**Fig 1:**
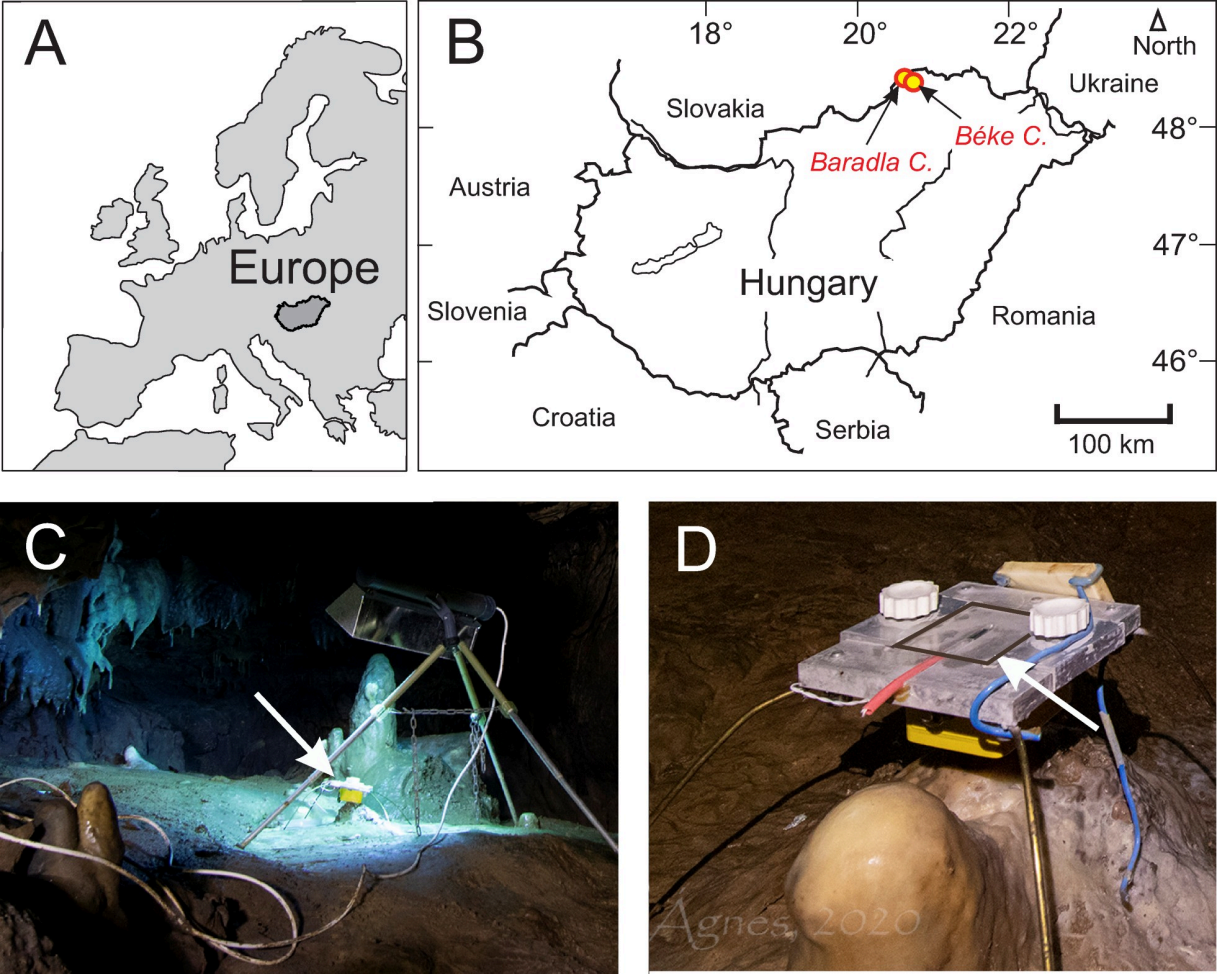
A) and B) Locations of the Baradla and Béke Caves. C) Sampling site illuminated with a UV-lamp. Arrow marks the position of glass plate holder. D) Glass plate holder under dripping water. Glass plate is contoured with a black line and marked by an arrow. Photos by Ágnes Berentés.

Monitoring activities and speleothem sampling were permitted officially by the Environment Protection Department, Miskolc District Office, Borsod-Abaúj-Zemplén County Government Office (permission No. BO-08/KT/4463-4/2017Miskolc). The activities were supervised by the Aggtelek National Park. The samples for the clumped isotope analyses consisted of the topmost surfaces of stalagmites from the Nehézút (NU) site and the surrounding area (Vaskapu, VK, about 100 m away) within the Baradla Cave, as well as two samples from the youngest part of a flowstone formation in the Béke Cave (see Fig 1 for locations). The stalagmites are described in detail by an earlier study [16].They represent compact stalagmites (NU-2 and VK-1) forming at a rate of about 0.3 mm year^–1^ and porous, more rapidly depositing (about 0.7 to 0.9 mm year^–1^) stalagmites (NU-1 and VK-2) [16]. The relatively high deposition rates, compared to the ~0.01 mm year^–1^ rate observed for stalagmites collected in the same cave [21], may be related to the stable drip water supply and to the nearly continuous dripping, which were important considerations when selecting the monitoring and stalagmite collection site [15,16]. The flowstone occurrence (drill core BNT-2) is approximately 5 km away from the Baradla Cave and has been extensively monitored [20] and geochemically analyzed [17]. Additionally, the carbonate produced by cultivated bacteria (*Rhodococcus degradans* strain BaTD-248 [18]) was treated with 5% NaClO solution to remove the agar medium used for cultivation. The treatment was conducted at room temperature in nitrogen atmosphere (to avoid artificial carbonate precipitation from air CO_2_) for one day, after which the sample was washed 10 times in distilled water and dried overnight at 40°C.

Glass plates were abraded on the collection side using F1000 grade silicon carbide (SiC) powder and were placed under dripping water at two sites (Fig 1) approximately 50 m away from each other at the Nehézút of the Baradla Cave [16]. The cave was monitored for two years [16,20] close to the carbonate farming sites, measuring local physico-chemical parameters (temperature, conductivity, pH, CO_2_ content) and drip water H and O isotope compositions, indicating that the site is stable and characterized by slight seasonal fluctuations, and no significant human influences on physico-chemical conditions. The temperature and CO_2_ concentration in the air of the cave show weak seasonalities (fluctuating between 10 and 10.5°C, and 2000 and 4000 ppm, respectively). In the current study, flying lead thermistor probes (Gemini Data Loggers Ltd.) were placed directly under the glass plates, and the temperature was continuously recorded by Tinytag Plus 2—TGP-4020 loggers manufactured by Gemini Data Loggers Ltd. The glass plates were situated on December 19, 2019 and were harvested and replaced on January 13, February 20, March 19, and May 29 in 2020. The glass plates were transported in a cooler box and stored in a refrigerator until analyses to avoid crystallization of amorphous calcium carbonate, if present. One site was illuminated using a double-ended UVC (germicidal) lamp emmitting light at 253.7 nm wavelengths, while the control site was left untreated. The lamp was repeatedly moved and placed in the former control site during sample collection to avoid site-specific bias.

To detect the germicidal effect of the UV lamp, two cultivation techniques were used to estimate and compare the number of viable microbes from the UV-treated and untreated habitats at all three sampling times. For indirect germ count estimation, a sterile cotton swab moistened with a sterile 0.9% (w/v) NaCl solution (pH 7.0) was vigorously scrubbed on the surface of the selected sample holder (approximately a 2 x 2.5 cm area) and immediately placed in a sterile tube containing 6 mL of sterile NaCl solution for transport to the laboratory. Contact TSA (DSMZ medium 535) plates were used for direct detection of viable microbes. The agar surface of the contact plate was pressed to the sample holders. Contact plates and samples collected in physiological saline were transported in a refrigerated container to the laboratory. For the latter sample, 10-fold dilution series were made using 4.5 ml of distilled water, and 0.2 ml of each dilution was plated onto R2A (DSMZ Medium 830) on the day of sampling. Samples from the original samples were also applied to the surface of the agar media in the form of droplets (200 μl-200 μl), without dilution. All plates were incubated at 20°C for 24 h, 48 h, and 1 week, before counting the number of colonies of culturable (untreated samples) and surviving (UV-treated samples) microbes. The results are expressed as colony forming units/cm^2^ (CFU).

Pieces of glass plates were broken, and their surfaces were coated with Au for scanning electron microscopy. The samples were analyzed using a Zeiss EVO 40 scanning electron microscope operated at 10 and 5 kV.

Scraped carbonate powder samples were analyzed by FTIR and micro-XRD at the Institute for Geological and Geochemical Research (IGGR) Budapest. Attenuated total reflection (ATR) FTIR analyses were performed using a Bruker Vertex 70 Fourier Transform Infrared spectroscope controlled by OPUS 8.1 software. For each sample, 16 scans were recorded in the 4000–400 cm^–1^ spectral range, with a resolution of 4 cm^–1^. Micro-XRD analysis was performed on a RIGAKU D/MAX RAPID II diffractometer, which is a unique combination of a MicroMax-003 third generation microfocus, sealed-tube X-ray generator, and a curved imaging plate (IP) detector. The diffractometer was operated with CuKα radiation generated at 50 kV and 0.6 mA. The powdered samples for the micro-diffraction measurements were encapsulated in a borosilicate-glass capillary, with a diameter of 0.3 mm and a wall thickness of 0.01 mm, by a vertical manual charging process. Then, the capillary was analyzed by the micro-diffractometer in transmission mode, with a beam spot diameter of 100 μm. For each measurement, 0.5–1 mg sample was placed in the funnel end of the capillary, and the sample was tapped into the narrow portion. The measurement took approximately 3 to 10 mins. The IP was read by a laser scanning readout system in approximately 1 min. 2DP RIGAKU software was used to record the diffraction image from the laser readout, allowing the operator to determine the area to integrate for a 2θ versus intensity plot. This plot was read into the RIGAKU PDXL 1.8 software for data interpretation.

Drip water samples were collected during the visits to the caves on January 13, February 20, March 19, and May 29 in 2020. Their stable oxygen isotope compositions were determined at the Institute for Geological and Geochemical Research using a liquid water isotope analyzer (type LWIA-24d) manufactured by Los Gatos Research Ltd. The instrument utilizes off-axis integrated cavity ring down spectroscopy to measure the absolute abundances of ^2^H^1^H^16^O, ^1^H^1^H^18^O, and ^1^H^1^H^16^O molecules via laser absorption [22]. The isotope compositions are expressed as δ^18^O values in ‰ relative to V-SMOW. Standardization was conducted using laboratory standard water samples that were calibrated to international standards [20]. The δ^18^O precision is better than ±0.15‰ (1SD).

Clumped isotope analysis of carbonate samples was run on a Thermo Scientific™ 253 Plus 10kV Isotope Ratio Mass Spectrometer (IRMS) in the Isotope Climatology and Environmental Research Center (ICER), Debrecen (Hungary). The phosphoric acid digestion of the samples was performed at 70°C with a Thermo Scientific™ Kiel IV automatic carbonate device, which is coupled to the IRMS by an inert silica coated capillary. The carbonate clumped isotope analysis system has a similar design to that described in previous publications [23–27]. Each carbonate sample measurement consisted of 10–12 replicate analyses of 100–120 μg aliquots that were divided into three measurement carousels (with 46 positions) and were measured alongside carbonate standard samples with assigned values. ETH1, ETH2, and ETH3 (S1 Table in S1 Appendix) were used as standard samples during the Δ_47_ calculation, and ETH-4 and IAEA-C2 (S1 Table in S1 Appendix) were used to monitor instrument performance. The applied δ^13^C, δ^18^O, and Δ_47_ values of these carbonate samples were published in Bernasconi et al. [28]. The ETH standards were used to transfer the sample Δ_47_ results into the absolute reference frame.

The Easotope application was used for data evaluation [29] with the CO_2_ clumped ETH PBL replicate analyses method and the IUPAC parameters, also called the Brand parameters [30–34] (S2 Table in S1 Appendix). A correction factor of 0.066 (Δ*25–70) [8] was applied to correct for different acid fractionation factors at 25 and 70°C and to represent Δ_47_ results on the CDES25°C scale. Further technical details of clumped isotope analyses and measurement data are provided in the Supplementary Materials (S1 Appendix, S1 and S2 Tables).

## Results and discussions

Cultivation and cultivation-independent analyses demonstrate that the surface of dripstones is inhabited by a diverse bacterial community [18,35], but their role in the precipitation of cave carbonates remains uncertain. Although a small percentage of bacteria can be cultivated from environmental samples [36], germ count estimation methods are usually applied to detect microbial contamination on surfaces.

**Fig 2:**
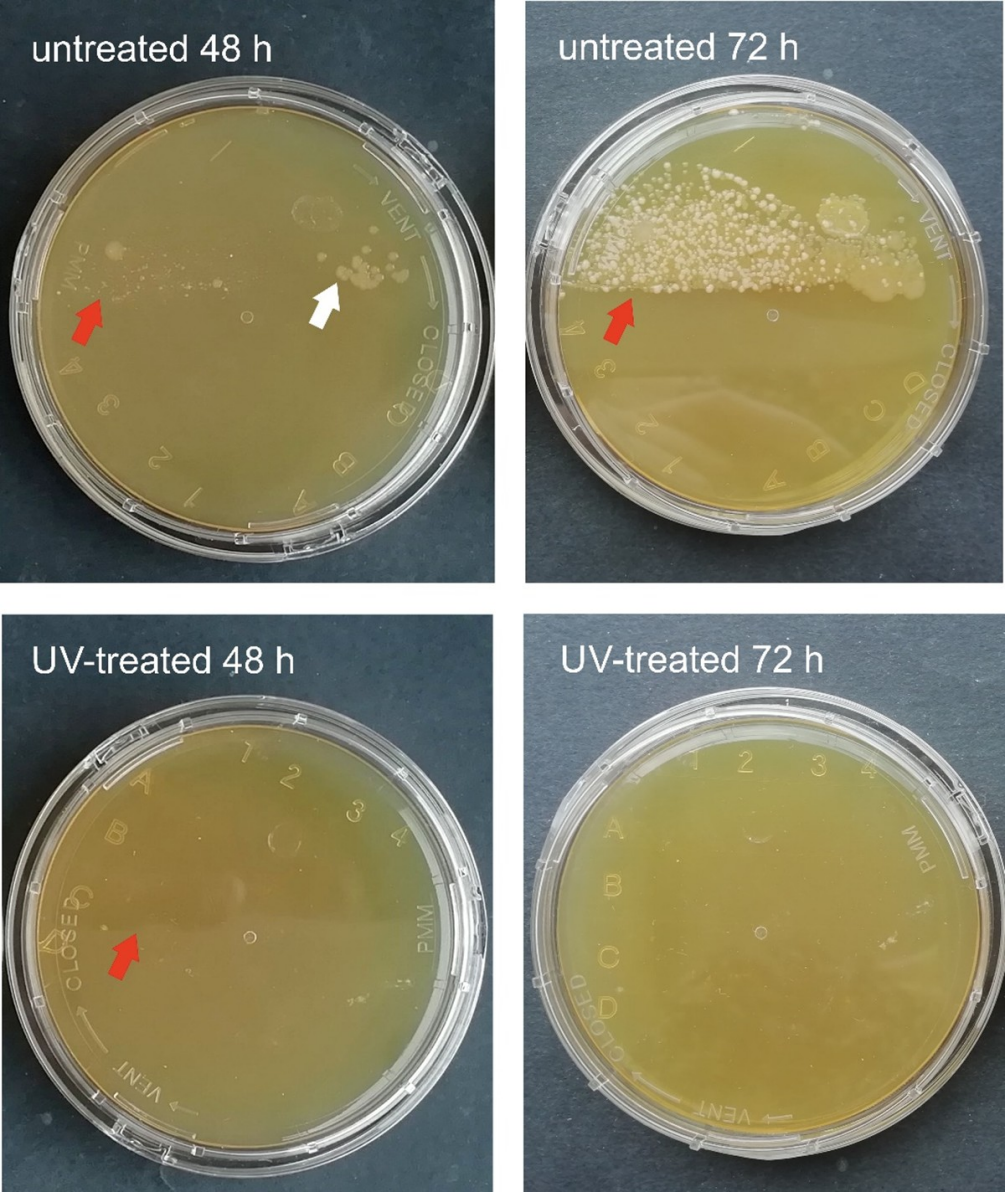
TSA contact plates inoculated with samples taken on May 29 after incubation at 20°C for 48 and 72 hours. Red arrows point to the edge of the part of the plate pressed to the sample holder. White arrows point to the bacterial colonies, which follow this edge from the untreated habitat.

**Fig 3:**
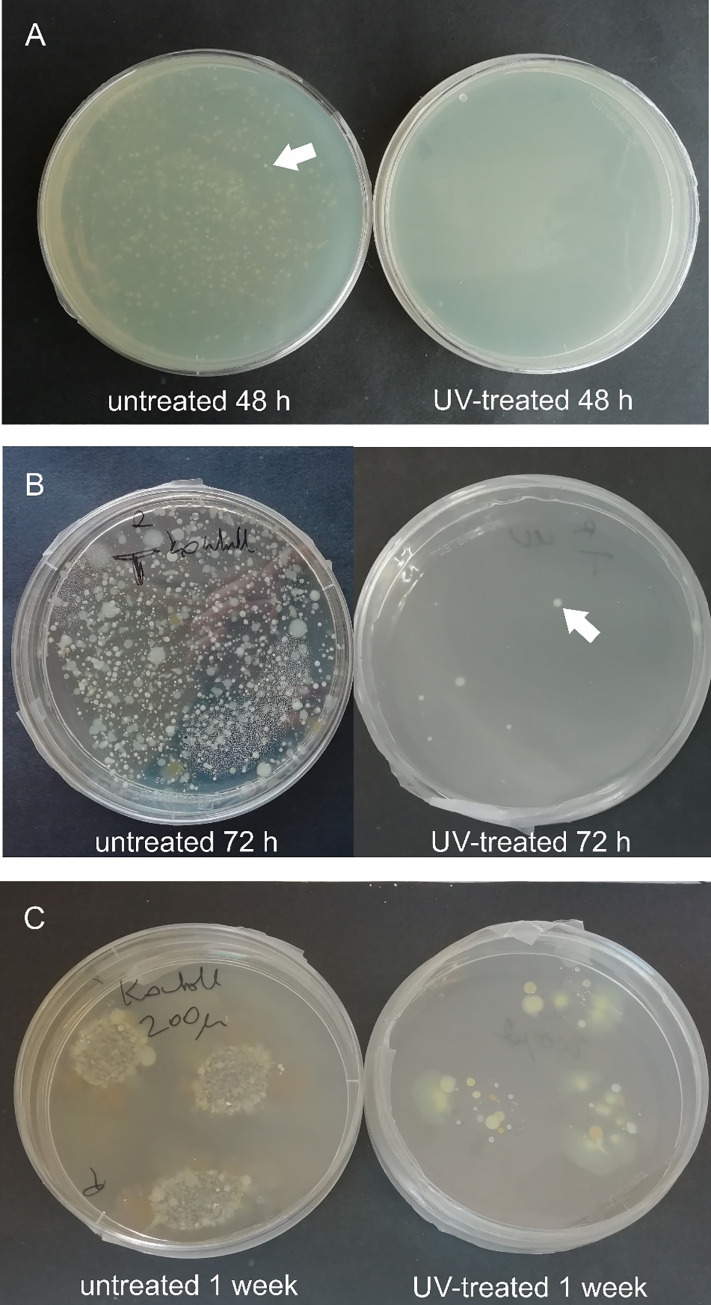
Agar plates inoculated with samples taken May 29 (A) and February 20 (B, C) after incubation at 20°C, for 48, 72 hours and one week. C panel shows plates inoculated with original samples in the form of droplets. White arrows point to the bacterial colonies.

A substantial difference was noted in the number of developing colonies incubated for 48 and 72 hours. A sporadic number of colonies was cultivated on R2A and TSA contact plates from the samples scrubbed from the UV-treated habitats, and high colony counts were observed on the surfaces of the untreated sample holders (Table 1, Figs 2 and 3). The colony counts differed depending on the length of time between samplings. The germicidal lamp prevented the bacterial cells in the drip water from surviving and proliferating. After one week of incubation, the number of colonies increased on the media inoculated with samples from UV-treated habitats, as well. This suggests that the vegetative forms of stress-related inactive bacteria (e.g., endospores) regenerated and multiplied on the more optimal, nutrient-rich medium. Bacteria isolated from subsurface environments also contain DNA repair mechanisms, which eliminate the damage caused by UV light [37,38]. During the sampling process, bacterial cells may land on the sample holders, thus suffering a low dose of UV irradiation. These cells may regenerate on the cultivation medium and form visible colonies.

**Table 1:** Stable carbon and oxygen isotope compositions (in ‰ relative to V-PDB) and clumped isotope data (CDES25°C) of the research samples in this paper.

sample	Comment	CFU/cm^2^	t °C	±	δ^18^Ow	n	δ^13^C	±1SD	δ^18^O	±1SD	Δ_47_	1SE	±95% CI
BNT-2/10	flowstone core, 10 mm from top		9.8	0.5	-9.4	21	-10.12	0.07	-6.84	0.12	0.730	0.007	0.015
BNT-2/top	flowstone core, topmost sample		9.8	0.5	-9.4	23	-9.82	0.05	-6.70	0.11	0.703	0.015	0.030
NU-1	fast growing stalagmite, top		10.2	0.5	-9.4	19	-10.37	0.06	-7.07	0.08	0.558	0.009	0.019
NU-2	slowly growing stalagmite, top		10.2	0.5	-9.4	18	-9.84	0.05	-7.13	0.08	0.547	0.011	0.024
VK-1	slowly growing stalagmite, top		10.2	0.5	-9.4	20	-10.54	0.05	-6.92	0.07	0.631	0.013	0.027
VK-2	fast growing stalagmite, top		10.2	0.5	-9.4	23	-10.49	0.06	-6.71	0.11	0.592	0.008	0.016
BaTD 248	cultivated bacterial carbonate		21	0.1		7	-20.36	0.07	-9.50	0.08	0.639	0.020	0.048
Glass plate 1	13/01/2020; site 2, untreated	1946	9.6	0	-8.5	16	-10.02	0.03	-7.31	0.06	0.679	0.008	0.018
Glass plate 2	13/01/2020; site 1, UV-treated	2	9.8	0	-8.1	11	-8.35	0.06	-6.83	0.08	0.769	0.011	0.026
Glass plate 3	20/02/2020; site 1, untreated	32107	9.8	0.1	-8.4	4	-11.90	0.05	-6.60	0.06	0.731	0.018	0.057
Glass plate 4	20/02/2020; site 2, UV-treated	33	10.4	0.2	-8.7	11	-10.97	0.05	-6.64	0.11	0.692	0.009	0.019
Glass plate 7	29/05/2020; site 1, UV-treated	48	10.0	0.5	-8.8	10	-11.12	0.10	-6.33	0.13	0.732	0.009	0.019
Glass plate 8	29/05/2020; site 2, untreated	>24000	11.0	0.3	-8.5	12	-11.70	0.07	-6.75	0.11	0.723	0.010	0.023

Temperatures (with uncertainties as total range) and water compositions (δ^18^Ow in ‰, relative to V-SMOW) were either obtained by monitoring during this study or were taken from earlier studies [15,20]. “n” is the number of replicate Δ_47_ sample measurements. Uncertainties of mean Δ_47(CDES25)_ are given as both: “±1SE” = one standard error of the mean (i.e., 1 SD/√n) and “±95% CI” = uncertainties at 95% confidence interval of population mean using a Student’s t-distribution.

FTIR and micro-XRD analyses show that the 1–2 months old carbonate samples collected from the glass plates of the control and UV-illuminated samples are dominated by calcite. These analyses did not detect ACC, contradicting the observation that this amorphous phase occurs in relatively fresh (1–7 days) cave carbonate precipitates [15] and on microbial colonies [18].

**Fig 4:**
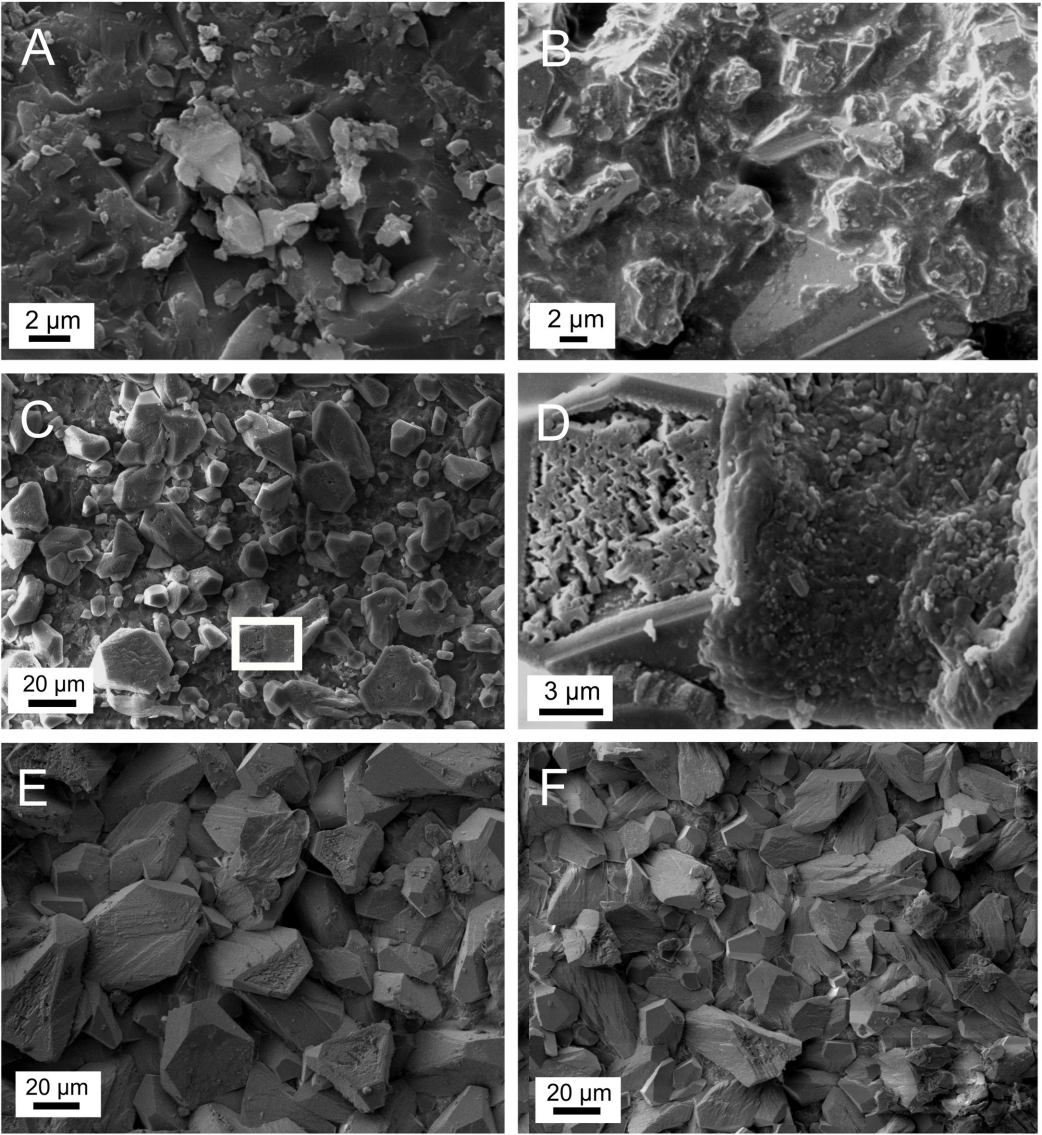
Scanning electron microscopic pictures of carbonates precipitated on glass plates. A) Glass plate collected on January 13, 2020, control site. B) Glass plate collected on January 13, 2020, control site, calcite crystals partially covered by biofilm carbonate. C) Glass plate collected on January 13, 2020, UV-illuminated site. The white rectangle marks the area of Fig 4D. D) Glass plate collected on January 13, 2020, UV-illuminated site. Left side: resorbed calcite crystal. Right side: biofilm carbonate with bacterial forms. E) Glass plate collected on May 29, 2020, control site. F) Glass plate collected on May 29, 2020, UV-illuminated site.

Microstructures and crystal forms of the control calcite samples farmed in January and February, 2020 indicate the effect of bacterial activity (Fig 4), particularly that the grain size is small (~ 1–3 micron, Fig 4A), the grains are covered by a biofilm (Fig 4B), and euhedral crystals are rare. In contrast, calcite particles of the UV-illuminated samples are well-crystallized, and the grains are larger (5–20 microns), relative to the control sample (Fig 4C). The pieces of biofilm carbonate on the surface of the illuminated samples (inside the white rectange in Fig 4C and shown at higher magnification at the right side of Fig 4D) can presumably be explained by dripwater transportation. Calcite dissolution is evidenced by pitted holes and rugged surfaces (e.g., Fig 4D, left side), whereas reprecipitation is indicated by the presence of small (micron-sized) calcite crystals inside the holes and on the rugged surfaces.

It is interesting that the morphology and structure of the control and the UV-illuminated samples farmed in May are similar (Fig 4E and 4F). The same size range (10–40 microns) and well-developed calcite crystals are observed in both samples, which are characteristic of abiogenic carbonates. The relatively large amount of dripwater likely gave rise to dominant inorganic calcite precipitation in May, thus, the relative amount of bacterial carbonate decreased compared to the carbonate collected in January and February.

**Fig 5:**
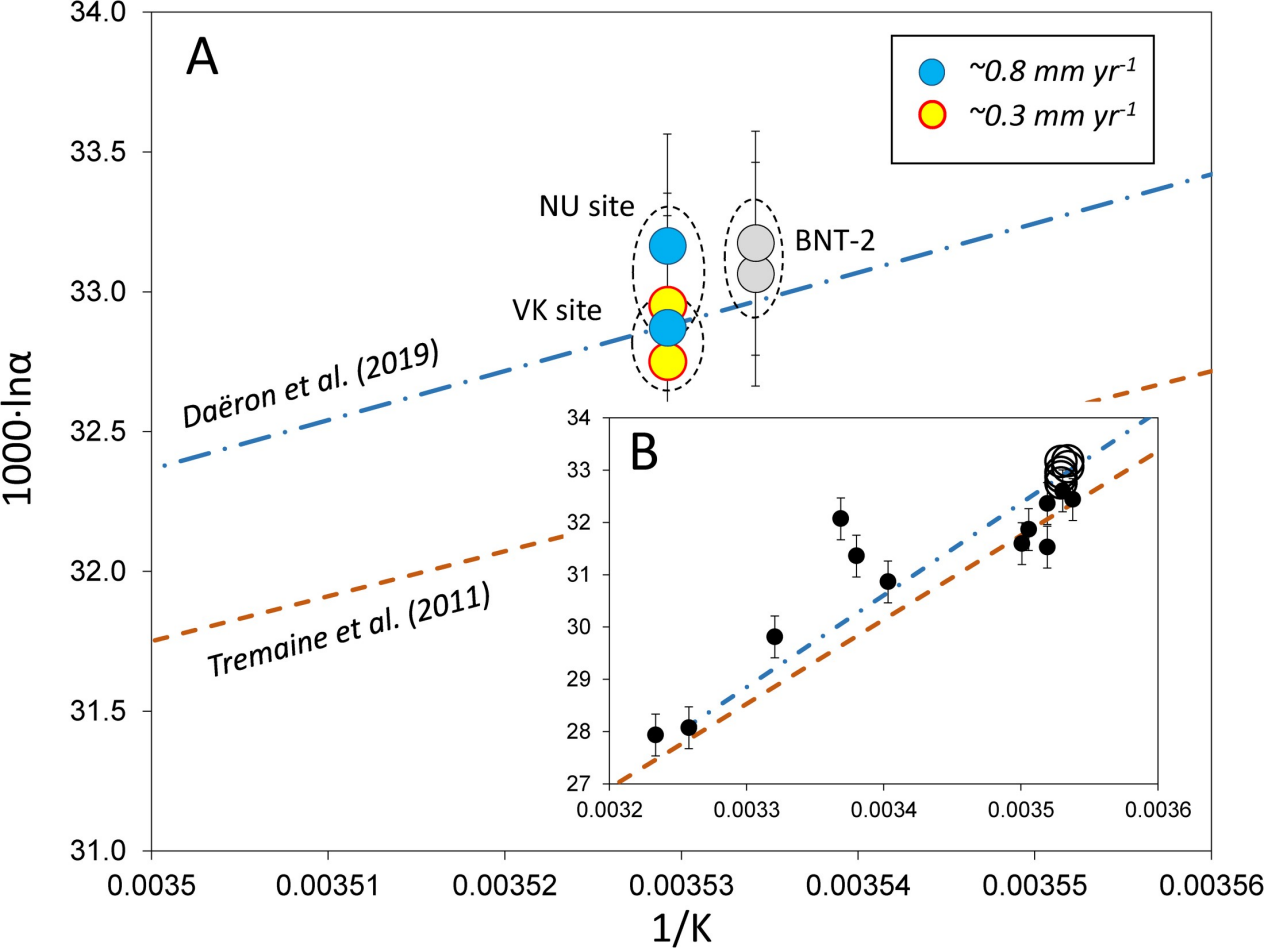
A) Calcite-water stable oxygen isotope fractionations of speleothems of the Baradla and Béke Caves. NU site: Nehézút, Baradla Cave, stalagmites NU-1, NU-2; VK site: Vaskapu site, Baradla Cave, stalagmites VK-1, VK-2 [16]; BNT-2: drill core of the Nagy-tufa flowstone [17]. Deposition rates are from Demény et al. [16]. B) Open circles: speleothems studied in this paper, dots: travertines [6]). Curves are from Daëron et al. [11] and Tremaine et al. [39], as in Fig 5A. Temperature uncertainties are smaller than the sample signs.

Equilibrium vs. kinetic fractionation during calcite precipitation is frequently evaluated by measuring the calcite-water oxygen isotope fractionation and formation temperature. The isotope fractionation is expressed by the 1000·lnα value, where α = (1000+δ^18^O_calcite_)/(1000+δ^18^O_water_). Drip water temperatures show small variations during the studied period at both sites (Site-1: T_min_ = 9.6°C, T_max_ = 11.4°C, T_average_ = 10.5°C; Site-2: T_min_ = 9.6°C, T_max_ = 10.0°C, T_average_ = 9.8°C), characterized by similar average values to those observed during previous long-term monitoring of this site (T_average_ = 10.2°C for the period of 1994–2013; [20]). Thus, formation temperatures and water compositions were obtained during monitoring activities, and the δ^18^O_calcite_ values were measured in this study (see Table 1). The 1000·lnα values are plotted in Fig 5 as a function of 1/T, where T is temperature in K. „Equilibrium” fractionation is represented by the curve of Daëron et al. [11], while the Tremaine et al. [39] curve, based on empirical observations for speleothems, is included for comparison (Fig 5A). The BNT-2 flowstone and the NU-1 and NU-2 stalagmite data are shifted to higher 1000·lnα values compared to the Daëron et al. [11] curve, whereas the VK stalagmites plot slightly below the „equilibrium” curve. It is interesting to note that the slowly depositing carbonates display lower fractionations than the faster precipitating ones. This suggests that an increased precipitation rate is associated with kinetic fractionation. The degree of kinetic effect is demonstrated in Fig 5B, where the travertine data of Kele et al. [6] are shown. Although the travertine data yielded a coherent Δ_47_-temperature relationship [6], their 1000·lnα values scatter widely compared to the stalagmites studied here.

**Fig 6:**
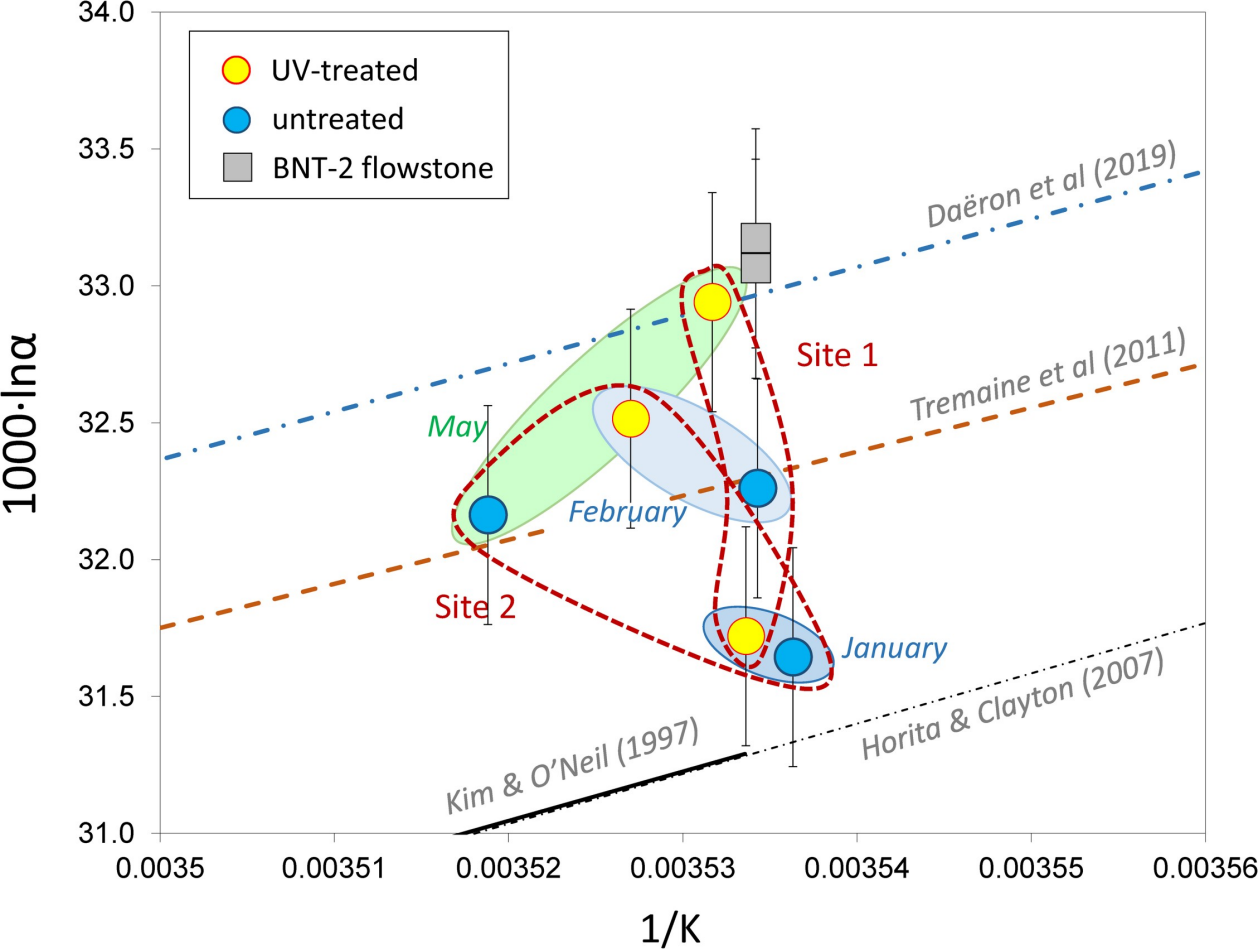
Calcite-water stable oxygen isotope fractionations of farmed calcites of the Baradla Cave. Empirical [11,39], experimental [40], and theoretical [41] curves are shown. Temperature uncertainties are smaller than the sample signs.

The farmed calcites scatter around the Tremaine et al. [39] curve. Systematic differences do not exist between the sites, and there is no relationship with the UV lamp treatment (Fig 6). Conversely, there appears to be a change in sampling period, as the data for the January sampling (with very low amount of deposited carbonate) differ to the February and May data, although it should be noted that the differences (~ ±0.6‰) are only slightly higher than the analytical uncertainty (~0.3‰).

**Fig 7:**
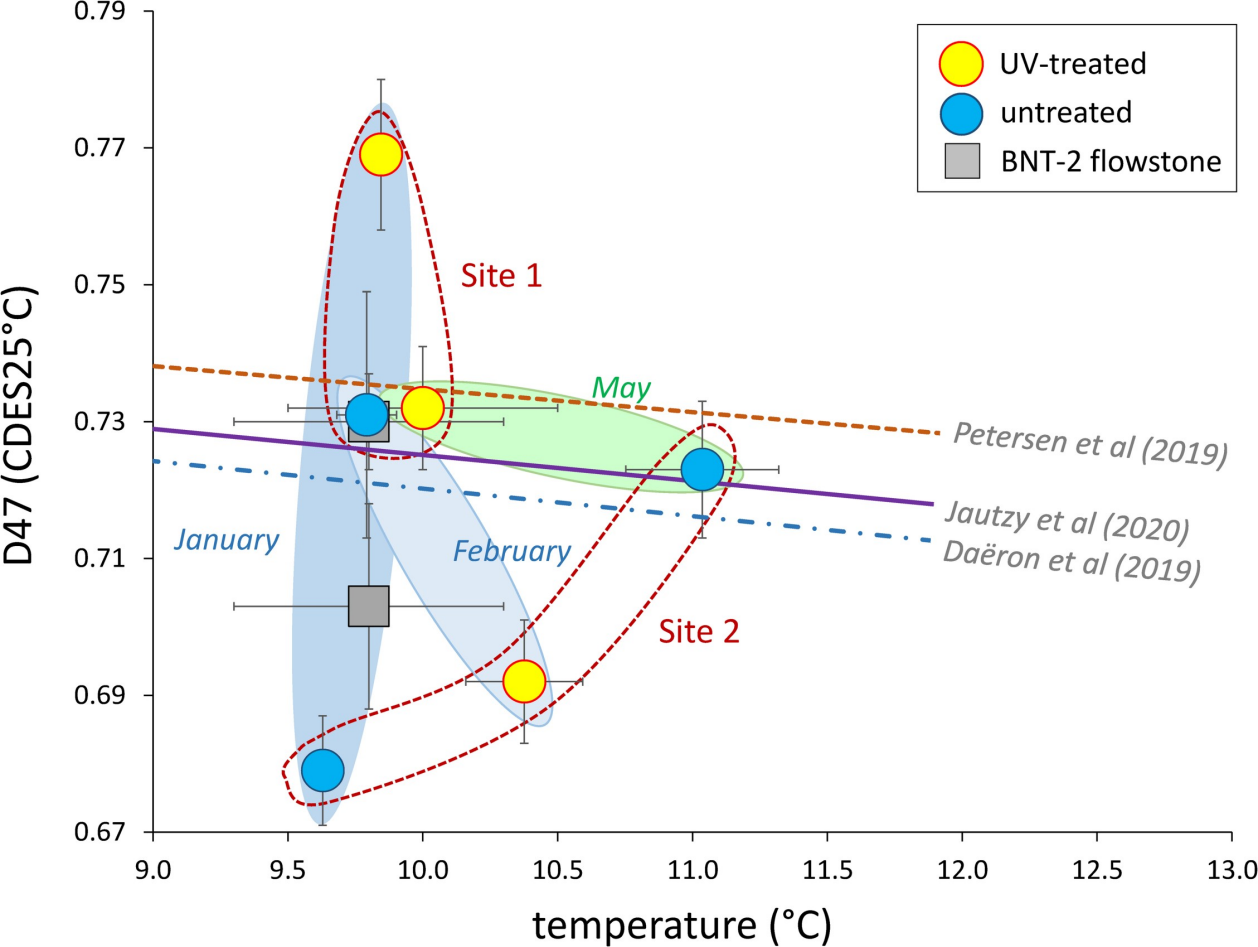
Clumped isotope compositions as a function of precipitation temperature for farmed calcites. Empirical and experimental „equilibrium” curves [8,9,11] are shown.

The calcite-water oxygen isotope fractionation values indicate that most of the speleothems and farmed calcites precipitated in close to isotopic „equilibrium”, with minor kinetic effects. However, the clumped isotope values of the NU and VK stalagmites are markedly lower than those of the BNT-2 flowstone data (Table 1), similarly to the published stalagmite data [13]. As the clumped isotope technique evolved significantly in the last decade, early data may not be compared with the present dataset, which was obtained following the correction scheme of Petersen et al. [8]. However, the isotope shifts due to methodology are minor compared to the differences between „equilibrium” Δ_47_ values [11] and stalagmite compositions [13]. The recent detection of ACC and its effect on the carbonate-water oxygen isotope fractionation [15], as well as evidence of the role of bacterial carbonate production in ACC formation [18] raise the question if microbially mediated ACC formation also affects clumped isotope compositions. The carbonate precipitated by the *Rhodococcus degradans* bacterial strain (BaTD-248) cultivated at 21°C yielded a low Δ_47_ value (0.639 ±0.018 SE) compared to the value (0.678) that would be given by the „equilibrium” curve [11], which may also support the existence of the bacterium-related vital effect. However, the farmed calcites precipitated under a UV lamp and in control conditons plot close to the „equilibrium” curve, with no systematic relationship to UV treatment (Fig 7). There appears to be a connection to the sampling period and the site, as the two sampling sites have slightly different compositional fields, and data for periods producing a small amount of farmed calcites (January and February) scatter more widely than the samples collected in May, when carbonate formation was more extensive (based on scrapable amounts of carbonate on the glass surfaces). It is important to note that calcite morphologies between UV-illuminated and control sites of the May collection do not differ significantly.

**Fig 8:**
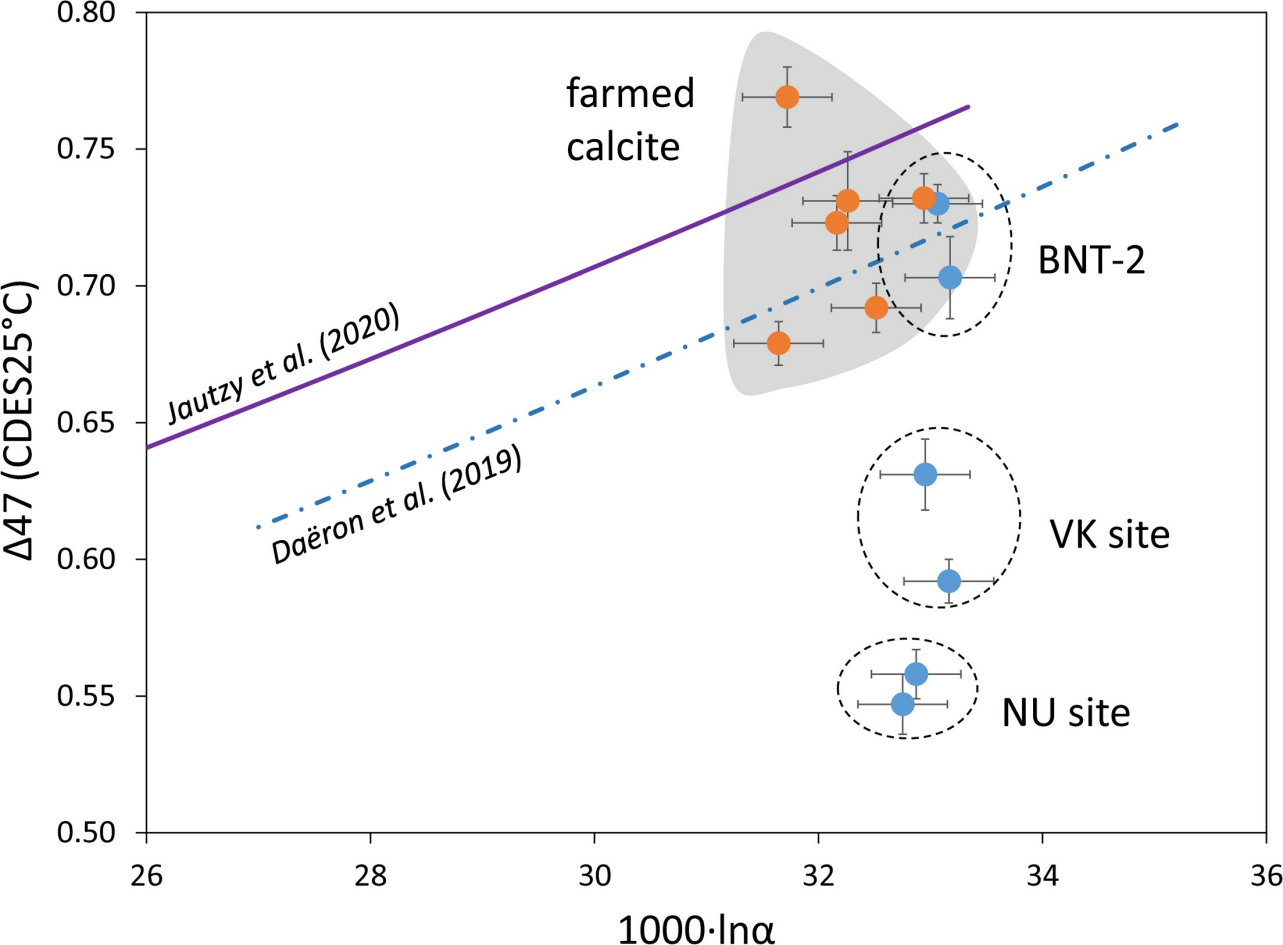
Clumped isotope compositions as a function of calcite-water stable oxygen isotope fractionation values for farmed calcites and speleothems.

The site effect is also pronounced in the case of speleothems. The farmed calcites (with one exception) and the BNT-2 flowstone data plot around the Daëron et al. [11] curve, whereas the stalagmites differ significantly (Fig 8). It should be noted that deposition rate plays a subordinate role to sampling site, as is exemplified by the NU stalagmites (NU-1: ~0.9 mm year^–1^; NU-2: ~0.3 mm year^–1^; [16]). The most important factors affecting clumped isotope compositions are likely kinetic fractionation along the drip waters’ seepage routes (degassing, evaporation, prior calcite precipitation), varying temporally and spatially between dripping points.

The close-to-„equilibrium” calcite-water oxygen isotope fractionation and the kinetically affected clumped isotope values can be related to different fractionation mechanisms. Disequilibrium Δ_47_ values of speleothems are explained by kinetic fractionation driven by CO_2_ degassing [e.g., 14,42] that may occur at the speleothem surface or along the seepage route. As the studied site is closed with no or very weak ventilation [20], extensive degassing and evaporation are not expected. Thus, the oxygen isotope exchange between DIC species and H_2_O results in δ^18^O values that are characteristic of non-kinetic speleothems [39]. In contrast, CO_2_ degassing may occur along the seepage route, and the clumped isotope disequilibrium might be inherited from DIC species, as suggested by a recent study [42]. This is indicated by the site-specific Δ_47_ shift described above.

## Conclusions

Speleothems and farmed calcites were analyzed for stable oxygen isotope and clumped isotope compositions to determine if bacterial carbonate production had a detectable affect on Δ_47_ values. Using a monitored site in the Baradla cave (NE Hungary) as a natural laboratory, calcites were deposited on glass plates under a UV lamp and in control conditions. Microbiological analyses showed that the UV treatment resulted in a significant decrease of viable count of bacteria on the glass plate. SEM analyses revealed significant morphological and microstructural differences between the control and UV-illuminated samples harvested in winter. FTIR and micro-XRD analyses determined that the deposited carbonate was calcite without an amorphous carbonate component. Compared to the UV-illuminated glass plates that showed well developed calcite crystals, the control samples collected in January and February showed textural features characteristic of bacterial carbonate. The calcite deposited in May was dominated by inorganic crystal forms, with no significant difference observed between the UV-illuminated and control sites.

Although most of the studied carbonates displayed δ^18^O values close to expected isotopic „equilibrium” value, stalagmites yielded disequibrium Δ_47_ values, while the farmed calcites and flowstone samples were characterized by close-to-„equilibrium” Δ_47_ data. Bacterial activity did not exert a detectable influence on clumped isotope compositions. The Δ_47_ values of speleothems were primarily determined by formation environment (precipitation from dripping water as stalagmite or from flowing water layer as flowstone) and site-specific kinetic processes related to degassing, evaporation, and prior calcite precipitation along the seepage routes.

## Supporting information

S1 Table Raw clumped isotope data of samples. (XLSX)

S2 Table Long-term raw data of standards used for clumped isotope analyses (ETH1, ETH2, ETH3, ETH4, IAEA-C2). (XLSX)

S1 Appendix Technical details of clumped isotope analyses including S1 and S2 Tables in S1 Appendix. (PDF)
