# Supplementary material for: Bacterial and abiogenic carbonates formed in caves–no vital effect on clumped isotope compositions
Source: PLoS One. 2021 Jan 25;16(1):e0245621. doi: 10.1371/journal.pone.0245621 (PMC7833151; doi:10.1371/journal.pone.0245621)
Supplement: S1 Appendix — (PDF) [file pone.0245621.s003.pdf]

S1 Appendix

Supplementary information for

“Bacterial and abiogenic carbonates formed in caves – no vital effect on clumped isotope composition”

Attila Demény[1,*], László Rinyu[2] , Péter Németh[3,4] , György Czuppon[1] , Nóra Enyedi[5] , Judit Makk[5] , Szabolcs Leél-Őssy[6] , Dóra Kesjár[2] , Ivett Kovács[1]

[1] Institute for Geological and Geochemical Research, Research Centre for Astronomy and Earth Sciences, Budaörsi út 45, Budapest, H-1112, Hungary

[2] Isotope Climatology and Environmental Research Centre (ICER), Institute for Nuclear Research, Bem tér 18/c, Debrecen, H-4026, Hungary

[3] Institute of Materials and Environmental Chemistry, Research Centre for Natural Sciences, Magyar tudósok körútja 2, H-1117 Budapest, Hungary

[4] Department of Earth and Environmental Sciences, University of Pannonia, Egyetem út 10, H-8200, Veszprém, Hungary

[5] Department of Microbiology, Eötvös Loránd University, Pázmány P. sétány 1/C, H-1117 Budapest, Hungary

[6] Department of Physical and Applied Geology, Eötvös Loránd University, Pázmány Péter sétány. 1/C, Budapest, H-1117, Hungary

*corresponding author email: demeny@geochem.hu

Contents

- Description of carbonate clumped isotope measurement
- Description of CO_2 clumped ETH PBL replicate analysis method with Easotope software

Additional Supporting Information (files uploaded separately)

- **S3 Table.** Raw clumped isotope data of samples.
- **S4 Table.** Long-term raw data of standards used for clumped isotope analyses (ETH1, ETH2, ETH3, ETH4, IAEA-C2).

Description of carbonate clumped isotope measurement

Clumped isotope analysis of carbonate samples was performed on a Thermo Scientific™ 253 Plus 10 kV Isotope Ratio Mass Spectrometer (IRMS) in the Isotope Climatology and Environmental Research Center (ICER) in Debrecen (Hungary). The phosphoric acid digestion of the individual samples occurred at 70 °C with a Thermo Scientific™ Kiel IV automatic

carbonate device, which was coupled by an inert silica coated capillary to the IRMS. To eliminate the organic contamination from the extracted carbon-dioxide gas an additional Thermo Scientific™ PoraPak trap was installed between the two cold fingers of the Kiel device. The temperature of the cold trap was controlled by a Peltier module. The trap was filled with a PoraPak™ Q 50-80 mesh porous polymer adsorbent, which was sealed at both ends with glass wool. The operation temperature of this trap was -30 °C. Generally, an hour-long 120 °C PoraPak trap cleaning and regeneration procedure is required before each measurement process. After the cryogenic purification the carbon-dioxide gas was measured against a working CO_2 gas (Linde AG, $\delta^{13}C_{V\text{-}PDB}$ = -3.9 ‰, $\delta^{18}O_{V\text{-}PDB}$ = -12.5 ‰, purity = 99.998%) for m/z 44-49 in micro-volume inlet mode and with long integration dual inlet (LIDI) method (Hu et al., 2014). The Thermo Scientific™ 253 Plus IRMS has 7 Faraday cups with resistors $3x10^{8}\Omega$, $3x10^{10}\Omega$ and $1x10^{11}\Omega$ for masses 44, 45, 46 and $1x10^{13}\Omega$ for masses 47, 47.5, 48, 49. The carbonate clumped isotope analyses system has a similar build-up to that described in previous publications (Schmid and Bernasconi, 2010; Hu et al., 2014; Meckler et al., 2014; Müller et al., 2017; Piasecki et al., 2019).

Each carbonate sample measurement consisted of several replicate analyses of 100-120 μg aliquots (normally 10-12) which were divided into three measurement carousels (with 46 positions) and measured together with carbonate standard samples with known values. ETH1, ETH2 and ETH3 were used as standard samples during the Δ_{47} calculation. In 2018 and 2019 ETH4 was used to monitor instrument performance. However, in 2020 IAEA-C2 took over the role of ETH4 as monitoring standard. The positions of the 46 aliquots in measurement carousel were the following:

1. 2 pcs ETH4 or IAEA-C2
2. 3 pcs ETH2
3. 4 pcs unknown sample 1
4. 3 pcs ETH1
5. 4 pcs unknown sample 2
6. 4 pcs ETH3
7. 4 pcs unknown sample 3
8. 3 pcs ETH2
9. 4 pcs unknown sample 4
10. 2 pcs ETH3
11. 4 pcs unknown sample 5
12. 3 pcs ETH1
13. 4 pcs unknown sample 6
14. 2 pcs ETH3

The applied $\delta^{13}C$, $\delta^{18}O$ and Δ_{47} values of these carbonate samples were published in Bernasconi et al. (2018) (S1 Table). ETH1, ETH2, ETH3 standards were used to determine the empirical transfer function and move the sample's Δ_{47} results into the absolute reference frame.

S1 Table. Applied values of ETH1, ETH2, ETH3, ETH4 and IAEA-C2 carbonate standards. Stable carbon and oxygen isotope compositions are given in ‰ relative to V-PDB. Clumped isotope value is given in CDES25 (carbon-dioxide equivalent scale unit).

Standard name	$\delta^{13}C$ V-PDB (‰)	$\delta^{18}O$ V-PDB (‰)	Δ_{47} (CDES25)
ETH1	2.02	-2.19	0.258
ETH2	-10.17	-18.69	0.256
ETH3	1.71	-1.78	0.691
ETH4	-10.20	-18.81	0.507
IAEA-C2	-8.25	-9.00	0.700

The signal collection using one replicate consists of 40 cycles in a 10 second integration period. During this collection period, the initial 17-18 V intensity of m/z 47 decreases to 12-13 V. The applied working gas was measured under the same conditions. The background correction of the Isodat NT software was disabled. Peak scans at six different intensities were used to calculate the pressure baseline correction values. The scans were performed before and after the measurement, and the correction values were interpolated (V44-V49 ETH PBL).

Data evaluation was carried out with Easotope software (Release 20190125, concept by Cédric John, programmed by Devon Bowen) (John and Bowen, 2016) using CO_2 clumped ETH PBL replicate analyses method and Brand parameters (Baertschi, 1976; Gonfiantini et al., 1995; Meijer and Li, 1998; Assonov and Brenninkmeijer, 2003; Brand et al., 2010) (S2 Table).

S2 Table."IUPAC" (International Union of Pure and Applied Chemistry) parameters and acid fractionation factor (AFF).

Parameter description	Value
Abundance ratio of $^{13}C/^{12}C$ for V-PDB	0.01118
Abundance ratio of $^{17}O/^{16}O$ for V-SMOW	3.8475E-4
Abundance ratio of $^{18}O/^{16}O$ for V-SMOW	0.0020052
Factor for converting $\delta^{18}O$ V-PDB for V-SMOW	30.92
Terrestrial mass-dependent fractionation parameter between ^{17}O and ^{18}O	0.528
Δ_{47} correction for acid fractionation factor (AFF) Δ^{*}_{25-70}	0.066

Description of CO_2 clumped ETH PBL replicate analysis method with Easotope software

Pressure baseline correction (V44-V49 ETH PBL)

Negative background effect, which is caused by secondary electrons, was eliminated by the utilization of a pressure base line correction (PBL). Peak scans at six different intensities (m/z 44: 5V, 10V, 15V, 20V, 25V and 28V) were used for PBL calculations. The high voltage region of these scans was 9.3909 – 9.6060 kV, with a 0.0005 kV step size, and the integration time was 0.1 sec (Meckler et al., 2014; Bernasconi et al., 2013). m/z 44 was used as a reference cup in all faraday cups for the PBL correction, which was based on regression (degree of fits is linear or quadratic), built from a selected region on the left side of the peaks (Meckler et al., 2014). Peak scans were performed before and after the clumped isotope measurement of the whole carousel (46 vials). The replicate was corrected by the interpolation of these two scan files. To determine the quality of the region selected, as well as the background correction, the average Δ_{47} value of ETH1 and ETH2 standards was compared. If the PBL was properly corrected, these values should be nearly identical. The built-in Δ_{47} nonlinearity correction was disabled during the calculation, as it over-corrected the background effect. Originally, the role of this step was to use standard measurements and a range of Δ_{47} and δ^{47} values to estimate the slope of the Δ_{47} vs the δ^{47} graphs. This method can determine the error caused by the nonlinearity of the ion-source. However, the PBL correction gives a better result for the correction calculation.

Absolute reference frame and empirical transfer function

Correction intervals of 3-4 weeks were applied in the Easotope software. This period depends on the stability of the mass spectrometer and/or the temperature stability of the mass spectrometer room. Typically, 45 individual standard measurements are considered for each sample calculation. ETH1, ETH2, and ETH3 were used to calculate the empirical transfer function (ETF). First, the software calculated the average Δ_{47} of each standard from the 45 individual replicates. Second, it plotted the average values in the Δ_{47} (WG) vs. the Δ_{47} (CDES70) coordinate space. Finally, the intercept and slope of the ETF was determined with the linear regression of these values. The ETF was used to transfer the sample Δ_{47} value into the absolute reference frame (CDES70). The ETF intercept and slope values, which were used for the calculation of the individual sample replicates, are presented in the attached "excel sheet with raw data of the samples" file, along with the measured isotopic compositions (S3 Table).

The identification of outlier data in the Δ_{47} population was accomplished by isolating the maximum number of different elements from the average value of the investigated population. An element was excluded from the population if the difference between its value and the population average was higher than 1.35 times the value of the standard deviation of the actual population. The population average and the standard deviation were recalculated. We applied this calculation repetitively. If the recalculated standard deviation was unchanged or higher than the previous value, the algorithm was completed, and the last excluded element added back into the population.

Acid fractionation factor

During the phosphoric acid digestion process, only two thirds of the carbonate oxygen is carried by the evolving CO_2 when the carbonate converted into carbon dioxide. This process results in oxygen isotope fractionation that depends on the reaction temperature. The full acid fractionation factor ($\Delta^{*}_{mineralogyX}$ for a given mineralogy and acid temperature X, as defined by Bonifacie et al., 2017) connects the measured Δ_{47} value of the extracted carbon dioxide and the Δ_{63} value in the original solid carbonate (Schauble et al., 2006). The temperature of the phosphoric acid digestion process is dependent on the laboratory's treatment method. To make the results of different laboratories comparable, a common carbon dioxide equivalent scale at 25 ºC (CDES25) was introduced. Instead of using $\Delta^{*}_{mineralogyX}$ to correct the carbon dioxide Δ_{47} to the carbonate Δ_{63} value, we used a fixed correction for the "acid fractionation factor" (AFF) to correct the measured Δ_{47} values to a reference reaction at 25 ºC. The applied treatment temperature in our Thermo Scientific™ Kiel IV automatic carbonate device was 70 °C. As such, Δ^{*}_{25-70} = 0.066 ‰ was used as the AFF in our calculations (Petersen et al., 2019).

Long-term stability of the standards

To determine the long-term stability of the system, the reproducibility of the used standards was calculated (details in S4 Table uploaded separately). In the case of ETH1, ETH2, and ETH3, the standard deviation was typically < 0.043‰ (1 SD), the standard error of the mean (1 SE) was 0.003‰, and the 95% confidence interval of population mean, using Student's t-distribution (95% CI), was < 0.006‰ for Δ_{47}. For ETH4, the reproducibility was < 0.064 ‰ (1 SD), the 1 SE was 0.010‰, and 95% CI was 0.019‰. Over the course 2020, we used IAEA-C2 as a monitoring standard, and the reproducibility for this standard was < 0.052 ‰ (1 SD), the 1 SE was 0.005‰, and the 95% CI was 0.011‰.

Supplementary references

Hu B, Radke J, Schlüter HJ, Heine FT, Zhou L and Bernasconi SM. A modified procedure for gas-source isotope ratio mass spectrometry: The long-integration dual-inlet (LIDI) methodology and implications for clumped isotope measurements. Rapid Communications in Mass Spectrometry. 2014; 28: 1413–1425.

Schmid TW and Bernasconi SM. An automated method for “clumped-isotope” measurements on small carbonate samples. Rapid Communications in Mass Spectrometry. 2010; 24: 1955–1963.

Meckler AN, Ziegler M, Millán MI, Breitenbach SFM and Bernasconi SM. Long-term performance of the Kiel carbonate device with a new correction scheme for clumped isotope measurements. Rapid Communications in Mass Spectrometry. 2014; 28: 1705–1715.

Müller IA, Fernandez A, Radke J, van Dijk J, Bowen D., Schwieters J, and Bernasconi SM. Carbonate clumped isotope analyses with the long-integration dual-inlet (LIDI) workflow: Scratching at the lower sample weight boundaries. Rapid Communications in Mass Spectrometry. 2017; 31: 1057–1066.

Piasecki A, Bernasconi SM, Grauel AL, Hannisdal B, Ho SzL, Leutert TJ, Marchitto TM, Meinicke N, Tisserand A and Meckler AN. Application of Clumped Isotope Thermometry to Benthic Foraminifera. Geochemistry, Geophysics, Geosystems. 2019; 0: 2082–2090.

Bernasconi SM, Müller IA, Bergmann KD, Breitenbach SFM, Fernandez A, Hodell DA, Jaggi M, Meckler AN, Millan I and Ziegler M. Reducing uncertainties in carbonate clumped isotope analysis through consistent carbonate-based standardization. Geochemistry, Geophysics, Geosystems. 2018; Vol 19., Issue 9: 2895-2914.

John CM and Bowen D. Community software for challenging isotope analysis: First applications of ‘Easotope’ to clumped isotopes. Geochemistry, Geophysics, Geosystems. 2016; Vol 30, Issue 21: 2285-2300.

Baertschi P. Absolute ^{18}O content of standard mean ocean water. Earth and Planetary Science Letters. 1976; Vol 31, Issue 3: 341-344.

Gonfiantini R, Stichler W, and Rozanski K (1995). Standards and intercomparison materials distributed by the International Atomic Energy Agency for stable isotope measurements. In Reference and Intercomparison Materials for Stable Isotopes of Light Elements. Vienna: International Atomic Energy Agency. 1995; IAEA-TECDOC-825, (pp. 13–29).

Meijer HAJ and Li WJ. (1998) The use of electrolysis for accurate $\delta^{17}O$ and $\delta^{18}O$ isotope measurements in water, Isotopes in Environmental and Health Studies. 1998; 34: 349-369.

Assonov SS, Carl S and Brenninkmeijer AM. A redetermination of absolute values for $^{17}R_{VPDB-CO2}$ and $^{17}R_{VSMOW}$, Rapid Com. in Mass. Spec. 2003; 17(10): 1017-29

Brand WA, Assonov SS, and Coplen TB- Correction for the ^{17}O interference in $\delta(^{13}C)$ measurements when analyzing CO_2 with stable isotope mass spectrometry (IUPAC Technical Report). Pure Appl. Chem. 2010; Vol. 82, No. 8, pp. 1719–1733.

Bonifacie M, Calmels D, Eiler JM, Horita J, Chaduteau C, Vasconcelos C, Agrinier P, Katz A, Passey BH, Ferry JM and Bourrand JJ. Calibration of the dolomite clumped isotope thermometer from 25 to 350 °C, and implications for a universal calibration for all (Ca, Mg, Fe)CO3 carbonates. Geochimica et Cosmochimica Acta, 2017; Vol. 200, 255–279.

Schauble EA, Ghosh G, and Eiler JM. Preferential formation of 13C-18O bonds in carbonate minerals, estimated using first-principles lattice dynamics. Geochimica et Cosmochimica Acta, 2006; Vol. 70(10), 2510–2529.

Petersen SV, Defliese WF, Saenger C, Daëron M, Huntington KW, Kluge T, John CM, Olack GA, Fiebig J, Lohmann KC, Passey BH, Peral MY, Petrizzo DA, Rosenheim BE, Tripati A, Venturelli R, Young ED, and Winkelstern IZ. Effects of Improved ^{17}O Correction on Interlaboratory Agreement in Clumped Isotope Calibrations, Estimates of Mineral-Specific Offsets, and Temperature Dependence of Acid Digestion Fractionation. Geochemistry, Geophysics, Geosystems. 2019; Vol. 20., 3495-3519.
